# Exosomes and exosome composite scaffolds in periodontal tissue engineering

**DOI:** 10.3389/fbioe.2023.1287714

**Published:** 2024-01-18

**Authors:** Tingyu Wang, Yanxing Zhou, Wenwen Zhang, Yuanye Xue, Ziteng Xiao, Yanfang Zhou, Xinsheng Peng

**Affiliations:** ^1^ The Second Affiliated Hospital of Guangdong Medical University, Dongguan, Guangdong, China; ^2^ Department of Pathophysiology, Guangdong Medical University, Dongguan, China; ^3^ Institute of Medical Technology, Guangdong Medical University, Dongguan, China; ^4^ Biomedical Innovation Center, Guangdong Medical University, Dongguan, China; ^5^ Institute of Marine Medicine, Guangdong Medical University, Zhanjiang, China

**Keywords:** stem cells, exosomes, tissue engineering, biomaterial scaffolds, periodontal regeneration

## Abstract

Promoting complete periodontal regeneration of damaged periodontal tissues, including dental cementum, periodontal ligament, and alveolar bone, is one of the challenges in the treatment of periodontitis. Therefore, it is urgent to explore new treatment strategies for periodontitis. Exosomes generated from stem cells are now a promising alternative to stem cell therapy, with therapeutic results comparable to those of their blast cells. It has great potential in regulating immune function, inflammation, microbiota, and tissue regeneration and has shown good effects in periodontal tissue regeneration. In addition, periodontal tissue engineering combines exosomes with biomaterial scaffolds to maximize the therapeutic advantages of exosomes. Therefore, this article reviews the progress, challenges, and prospects of exosome and exosome-loaded composite scaffolds in periodontal regeneration.

## 1 Introduction

Periodontal tissues are a complex and dynamic system comprised of both hard and soft tissues that envelop and uphold teeth. The hard tissues, namely, dental cementum and alveolar bone, work synergistically with the soft tissues, specifically the periodontal ligament and gingiva, to effectively withstand and distribute the forces generated during mastication. This intricate interplay between hard and soft tissues is essential for maintaining the integrity and functionality of the periodontium (Bartold et al., 2006). Periodontitis is a chronic inflammatory disease that occurs in periodontal tissues and is caused by an imbalance in the microbiota surrounding the periodontal tissues. Periodontitis is characterized by an imbalance or relative abundance of microbial species. Chronic inflammation can cause increasing damage to periodontal tissue and eventual tooth loss over time. Moreover, the damage caused by periodontitis to periodontal tissues is irreversible. In periodontal disease, a dysbiotic and destructive host response is induced by a polymicrobial community through a mechanism of microbial synergy and dysbiosis. The primary cause of osteoclast activation and alveolar bone resorption is an overactive host immunological response (Lamont et al., 2018). Severe periodontitis not only causes tooth loss, but it also raises the risk of arteriosclerosis, poor pregnancy outcomes, Rheumatoid Arthritis, aspiration pneumonia, and cancer, all of which have a negative impact on overall health (Hajishengallis, 2015).

In the early stages of periodontitis, slight regeneration of periodontal tissues may be observed. Only treatment intervention, however, can stimulate regeneration once periodontitis has been detected. Traditional periodontal treatment mainly includes supra-gingival scaling, sub-gingival curettage, and root planning to remove pathogenic factors, supplemented with systemic or local antibiotics or other therapeutic drugs to optimize treatment (Wang et al., 2022). However, these methods can only prevent further inflammation and cannot repair damaged periodontal tissues. Emerging treatment approaches such as guided tissue regeneration (GTR), autogenous bone grafting, and allograft bone filling have enriched traditional methods of periodontal treatment over the past few decades and may achieve some periodontal regenerative effects. However, there are still problems such as limited applicability, high clinical technical sensitivity, secondary inflammation and immune rejection of periodontal tissues, and unclear treatment effects. The development of tissue engineering provides the possibility for periodontal tissue regeneration and repair. Tissue engineering is a multidisciplinary field that integrates principles from cell biology, materials science, and biomedical engineering to fabricate biological substitutes capable of emulating or replacing the function of damaged or diseased tissues. Stem cell composite scaffolds are now widely used in tissue regeneration, but their clinical application is limited due to difficulties in extraction, storage, limited quantity, and unavoidable problems such as immune rejection and ethics. Exosomes, as a prominent byproduct of stem cell paracrine signaling, exert a pivotal role in tissue regeneration following injury. These extracellular vesicles, enriched with a diverse array of proteins, lipids, and nucleic acids, serve as critical mediators of intercellular communication while exhibiting favorable traits of biological safety, stability, and efficient delivery. Numerous studies have identified exosomes as a natural nanoparticle delivery method that can treat a number of inflammatory, immunologic, and infectious diseases (Kibria et al., 2018; Akbar et al., 2022). Exosomes have now become a very promising noncellular therapy, and using biomaterial scaffolds to load exosomes can achieve superior therapeutic effects. On this basis, this paper reviews the research progress of non-cell-based tissue engineering therapy in periodontal regeneration and discusses its shortcomings and prospects for further development.

As a chronic inflammatory disease, periodontitis is expected to improve local inflammation and promote periodontal tissue regeneration by exosome therapy. Nowadays, various exosomes derived from stem cells have been applied in the field of periodontal tissue engineering, and achieved good effects in controlling periodontal tissue inflammation and promoting alveolar bone regeneration, by loading exosomes with biomaterial scaffolds, exosomes can be released slowly in the injured area to maximize the advantages of exosomes. In summary, this paper aims to discuss the progress of cell-free periodontal therapy based on tissue engineering, and discuss its shortcomings and prospects.

## 2 Extracellular vesicles

Extracellular vesicles (EV) are natural envelope nanoparticles secreted by all types of cells, including prokaryotes and eukaryotes. They are important mediators of cell-to-cell communication in the form of proteins, lipids, nucleic acids, and even organelles, which are transmitted to surrounding or distant cells to transmit information (Radler et al., 2023). Studies have shown that extracellular vesicles contain a range of proteins, sugars, lipids, and genetic materials including DNA, mRNA, and non-coding RNA. These vesicles are not influenced by extracellular space protease and nuclease (Henderson and Azorsa, 2012). In general, extracellular vesicles can be classified into three distinct types based on their origin: 1) exosomes (50–150 nm) which are produced in the lysosome and plasma membrane (Pegtel and Gould, 2019; Fordjour et al., 2022); 2) microvesicles, which originate directly from the plasma membrane (0.1–1 μm) (Pollet et al., 2018); and 3) apoptotic bodies (1–5 μm) that are formed by deceased cells (Li et al., 2020). Macrophages can recognize and eliminate apoptotic bodies. Exosomes and microvesicles are currently the most extensively researched. The medical community has shown a keen interest in exosome-based cell-free therapy in recent years. Therefore, this review primarily focuses on the history, composition, and functions of exosomes, along with their potential applications in tissue engineering.

## 3 Exosomes

### 3.1 Biogenesis and composition of exosomes

Exosomes were originally found in the supernatant of sheep erythrocytes cultured *in vitro* (Pan and Johnstone, 1983). They were microvesicles with a diameter of 40–150 nm (Trubiani et al., 2019), found in a variety of cells (such as stem cells, tumor cells, epithelial cells and nerve cells) and body fluids (such as saliva, milk, amniotic fluid, serum or, plasma and urine, etc.) (Johnstone et al., 1987; Kalluri and LeBleu, 2020). The endosomal sorting complex required for transport (ESCRT), located on the cytoplasmic side of the endosome, is a protein complex of the endosome, it can encapsulate protein, nucleic acid and so on, and form multivesicular body containing vesicles (Hessvik and Llorente, 2018). However, when ESCRT is depleted, the formation of multivesicular bodies is assisted by the action of Ceramide, transmembrane proteins, etc. Subsequently, multivesicular bodies rely on the Rab family to control the transport of intracellular vesicles, localize vesicles to the cell membrane, and, under the regulation of the SNARE family (Mashouri et al., 2019), promote vesicle membrane fusion to the plasma membrane after the fusion of the vesicle membrane to the cell membrane; Exosomes are secreted outside the cell (Bache et al., 2003; Hanson and Cashikar, 2012).

**FIGURE 1:**
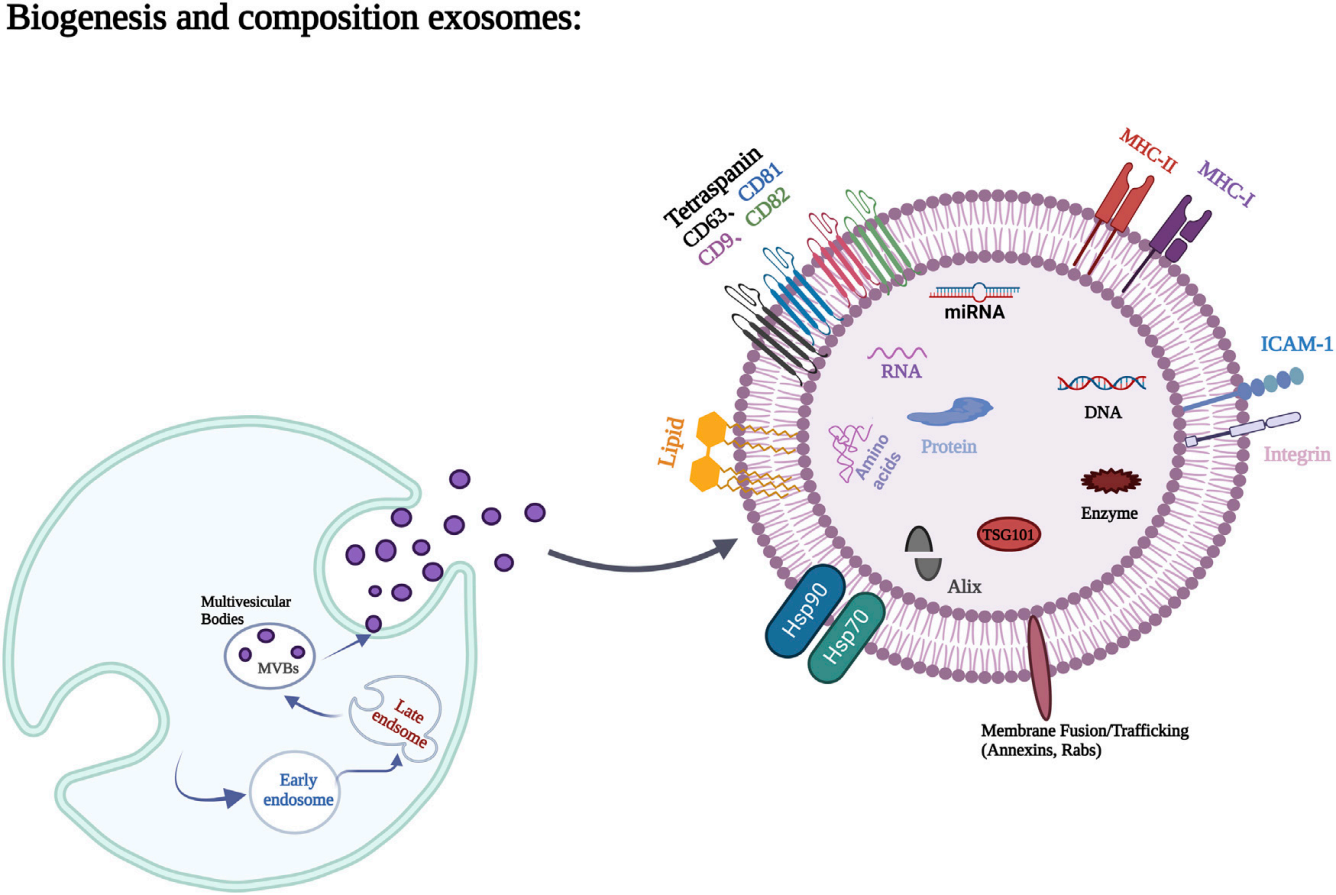
Biogenesis process and main components of exosomes. Figures created with BioRender.com with an academic license.

Exosomes contain a variety of proteins, lipids, RNA, miRNA, and many other non-coding RNA inside and on their surfaces (Mathivanan et al., 2010). Proteins occupy the majority of extracellular vesicle contents and are mainly composed of non-specific proteins and specific proteins (van Niel et al., 2006). Adhesion molecules such as cell adhesion molecules, integrins, tetracycline, lymphocyte and dendritic cell MHC I, class II molecules, and transferrin receptor (TFR) on the surface of Reticulocyte are typically specific exosomal proteins (Deng and Miller, 2019). Membrane fusion and transport proteins, such as Annexin, Flotillin, GTPases, Rab2, Rab7, Heat shock protein proteins, such as HSP70, HSP84, HSP90, transmembrane proteins, such as CD9, CD63, CD59, as well as actin, myosin, the cytoskeleton proteins such as tubulin, and the non-specific proteins such as Alix, which mediate the formation of MVB, belong to exosomes (Poliakov et al., 2009; Mashouri et al., 2019). These non-specific proteins play an important role in the production and release of exosomes. Lipids are also important components of exosomes, which are usually rich in cholesterol, glycosphingolipids, Ceramide and phosphatidylserine. Lipids not only play a structural role in exosome membranes, but also play an essential role in the formation and release of exosomes into the extracellular environment (Skotland et al., 2019). The biogenesis of exosomes and the major components of exosomes are shown in Figure 1.

### 3.2 Extraction and identification of exosomes

Currently, diverse methodologies for the isolation and purification of exosomes have been devised, which are predicated on the biophysical characteristics, such as density, size, and morphology, of exosomes, as well as their biochemical properties, including surface antibodies and cellular membranes. Efficient isolation strategies enable the precise quantification of exosomal signals while circumventing the confounding presence of lipoproteins, non-vesicular protein aggregates, and other extracellular vesicles that resemble exosomes in size and density, thereby thwarting contamination (Lin et al., 2022).

The current exosome separation techniques primarily comprise: 1) Separation technology utilizing ultracentrifugation, which encompasses differential ultracentrifugation and density gradient ultracentrifugation. Ultracentrifugation, the earliest, most classic, and commonly used isolation method for exosomes, is simple and does not require the introduction of additional markers. This method is appropriate for high-dose sample analysis (Yu et al., 2018). However, the method’s performance under centrifugal forces of up to 100,000 × g can generate diverse vesicles and protein aggregates of comparable size. Therefore, exosomes prepared using this method necessitate additional purification (Van et al., 2012). Moreover, high-speed centrifugal forces might disrupt exosome structures, significantly impacting downstream experiments. Density gradient ultracentrifugation is an improved method based on ultracentrifugation that makes use of the difference between particle density and medium density to make different particles under certain relative centrifugal forces, a specific point of concentration in a gradient medium. Finally, various high-purity particles can be collected from different areas. This method can separate the particles with lower precipitation coefficient, and the particles keep moving in the process of gradient centrifugation without squeezing or deformation. However, this method is cumbersome and time-consuming. 2) Separation technology based on size screening-ultrafiltration and size exclusion chromatography. The main principle of ultrafiltration is to use a membrane with a specific pore size or cut-off molecular weight to separate particles in the corresponding size range (Xu et al., 2017), but the membrane is expensive and easily blocked, so the cost is large. For size exclusion chromatography, the principle is to use an initial biological liquid as the mobile phase and a porous gel-folded polymer as the stationary phase (Ing et al., 2014), the properties of which allow for the elution of different particles. The larger particles are eluted first, followed by smaller vesicles, and finally non-membrane-bound proteins (An et al., 2018), with different-sized particles in different stages of elution. However this method also has some disadvantages such as the existence of similar size pollutants and low separation efficiency. 3) Separation technology based on immunoaffinity capture is a method to separate exosomes according to the principle of antigen-antibody specific recognition and binding. Exosomes isolated by this immune technique contain at least twice as many exosome markers and proteins as those by superfluous ultracentrifugation, and exosomes isolated by this method have higher specificity and yield (Sander et al., 2016). However, the need for more expensive antibodies to handle large samples can only be applied to small sample studies, which to some extent limits its large-scale use. 4) Polymer precipitation separation technology is a method that can create a hydrophobic microenvironment by hijacking water molecules around exosomes (Zeringer et al., 2015), thereby forcing exosomes to leave solution and sediment at low speed centrifugation. This method has the advantages of high yield and low cost, and the obtained exosomes are not deformed. However, the purity of exosomes obtained by polymers is low, and there is co-precipitation of soluble non-exosomal proteins, virions, Immune complexs and other pollutants (Pin et al., 2017). 5) Microfluidic separation technology includes size-based microfluidic separation technology and immune microfluidic technology, and size-based microfluidic separation is a technique in which modular units of membranes with different pore sizes are connected to separate exosomes of a specific size range. The exosomes isolated by this method are simple in structure and easy to use, and can achieve high recovery and separation purity with a small sample size (10–100 uL) (Liu et al., 2017). Immune microfluidic technology is a label-based separation technique similar to immune affinity capture methods. Exosomes are isolated by binding fused antibodies to exosome markers on a microfluidic device or chip. The technique offers advantages such as high efficiency, fast processing speed, simple operation, and suitability for small sample sizes. However, the purity of the resulting exosomes requires further improvements. 6) Aptamer-based isolation techniques, because aptamers are short single-stranded DNA or RNA sequences, are able to recognize and bind their targets in an antibody-like manner and have high safety and specificity. Compared with traditional antibodies, aptamers can be synthesized *in vitro* and have the advantages of low immunogenicity, low cost and easy chemical modification (Wang et al., 2019a; Wang et al., 2019b).

The identification of exosomes primarily involves the identification of morphological features and protein composition. Techniques for morphological identification encompass a range of methods, including scanning electron microscopy, transmission electron microscopy, dynamic light scattering, and nanoparticle tracking analysis. Protein composition identification methods include Western blotting, BCA protein detection, and flow cytometry.

It is difficult to obtain 100% pure exosome samples by existing exosome isolation techniques, and although the purity of exosomes can be improved by combining different isolation techniques, they usually increase the difficulty and cost of operation and may lead to a decline in total output and unreliable downstream analysis. Therefore, before choosing an isolation strategy, it is necessary to carefully consider the nature of the sample and the purpose of the study to select the appropriate technology combination (Liu et al., 2022). At the same time, there is no single method to analyze the biophysical and biochemical properties of exosomes in isolated samples. Combining two or more methods to improve the yield, purity and efficiency of foreign proteins is a feasible strategy.

## 4 Exosomes in periodontal tissue repair

Exosomes harbor a diverse array of biomolecules, comprising proteins, messenger RNA (mRNA), microRNA (miRNA), free fatty acids, surface receptors, and cytokines, among others. Given their cargo, exosomes can serve as agents for both local and systemic intercellular communication (Meldolesi, 2018). The function of exosomes is closely related not only to their cell source but also to the types of proteins and RNA they contain. Exosomes from different sources have different roles in various physiological and pathological stages. As shown in Table 1, the effects of exosomes from different cell sources on periodontal inflammation and immunomodulatory factors, therapeutic efficacy and related mechanisms are reviewed. Exosomes encompass an array of transmembrane proteins, including CD9, CD63, CD81, and CD82, along with heat shock proteins, lipoproteins, and diverse transport-related proteins. These constituents not only facilitate the identification of exosomes as foreign entities but also endow them with the capacity to selectively target recipient cells (Thery et al., 2002). Exosomes can be internalized by recipient cells via autocrine or paracrine signaling pathways and can disseminate to target tissues or organs via the circulatory system, thereby participating in diverse physiological and pathological processes that support tissue repair. Furthermore, exosomes play an essential role in preserving tissue homeostasis via both short- and long-range intercellular communication within tissues (Huang et al., 2021). Numerous studies have demonstrated that miRNAs contained within exosomes possess the ability to induce biological responses in recipient cells. For example, hMSC exosomes exert miRNA-dependent control that contributes to osteoblastic differentiation (Shirazi et al., 2021). Additionally, exosomes can directly act on the cell surface and trigger repair processes in recipient cells by activating intracellular signaling pathways (Roefs et al., 2020). In addition, exosomes have the advantages of higher biological stability, immune privilege, and more convenient delivery. Exosomes, as a novel cell-free therapeutic strategy, have been utilized as a substitute for mesenchymal stem cells in various disease models, including neurological, cardiovascular, immunological, renal, musculoskeletal, hepatic, respiratory, ocular, and cutaneous diseases, as well as cancer (Chen et al., 2019).

**TABLE 1:** Overview of the effects, therapeutic efficacy, and related mechanisms of exosomes from different cell sources on inflammation and immune regulatory factors in periodontitis.

Cell sources of exosomes	Effects in the treatment of periodontitis	Related mechanisms	Therapeutic efficacy	References
Dental pulp stem cells (DPSCs)	1. Decreased gingival inflammation and immune cell infiltration in periodontitis mice	Nfat5 was dramatically downregulated by the miR-1246 found in the exosomes of dental pulp stem cells because it reduced the development of Th17 cells and restored the Th17 cell/Treg balance in periodontal tissue	Reduced inflammation of periodontal tissue	Zhang et al. (2021a)
	2. Downregulated the expression of Il-1β, Il-1α, TNF and Ccl12			
	3. Restoring the balance of Th17 cells/Treg and enhancing the anti-inflammatory effect			
Dental pulp stem cells (DPSCs)	1. Il-23, Il-1, TNF-, Il-12, Il-1, Il-27, and Il-17 concentrations in periodontal tissues of mice with periodontitis were reduced	1. DPSC-Exo downregulated the NF-κB p65 and p38 MAPK signaling pathways in periodontitis mice, alleviating periodontal inflammation	Healing of alveolar bone and periodontal epithelium in periodontitis mice	Shen et al. (2020)
	2. Promote the transformation of macrophages from pro-inflammatory phenotype to anti-inflammatory phenotype	2. The miR-1246 in DPSC-Exo promotes the transformation of macrophages from pro-inflammatory phenotypes to anti-inflammatory phenotypes		
Human leukocyte antigen (HLA) haplotype homologous dental pulp cells (HHH-DPCs)	1. TNF- and IL-17, two pro-inflammatory cytokines, were secreted less frequently	The immune system may be involved, such as macrophages, osteoclast precursors, and T cells	Alveolar bone revealed a preservation effect from HHH-DPC exosomes	Shimizu et al. (2022)
	2. Enhances the production of the anti-inflammatory molecules Il-10 and TGF- and encourages the differentiation of CD4^+^ T cells into regulatory T cells			
	3. Inhibit the formation of osteoclasts			
Reparative m2-like macrophages	1. The expression of osteoclast related genes such as ACP, NFATC1, c-Fos and MMP-9 decreased	*In vitro*, exosomal IL-10 mRNA is delivered directly to cells, and IL-10 cytokine expression is upregulated in BMSCs and BMSCs, activating the IL-10/IL-10R pathway and regulating cell differentiation and bone metabolism	Decreased alveolar bone resorption in periodontitis mice	Chen et al. (2022)
	2. Osteogenesis-related genes Alp, COl1a, Runx2 and Ocn were upregulated			
Periodontal ligament stem cells (PDLSCs)	PDLSC exosome treatment resulted in the production of mineralized nodules as well as enhanced expression of osteogenic genes (Runx2 and Ocn) and proteins in periodontitis periodontal ligament stem cells (i-PDLSCs)	It blocked the over-activation of the classical WNT signal and restored i-PDLSC osteogenic differentiation	Promote bone regeneration in alveolar bone defects	Lei et al. (2022)
Human gingival mesenchymal stem cells	Pro-inflammatory M1 macrophages transformed into anti-inflammatory M2 macrophages: reduced expression of IL1, TNF-and iNOS; increased expression of CD206	Exosome miR-1260b inhibits osteoclastogenesis through Wnt5-mediated RANKL pathway	Reduces inflammation and prevents bone loss in periodontal tissue	Nakao et al. (2021)
Dental follicle cells (DFCs)	1. Encourage periodontal cell proliferation, migration, and differentiation	In rats with experimental periodontitis, the OPG/RANK/RANKL signaling pathway is partially mediated to reconstruct and regenerate periodontal tissue	Favorable restoration of alveolar bone lost in early stages of treatment in rats with experimental periodontitis and maintenance of alveolar bone level in later stages of treatment	Shi et al. (2020)
	2. Increases the expression of genes associated with osteogenesis, such as OPN, OCN, and Runx2			
	3. Increase COL I, CMEP-1, fibronectin, and Periostin expression in periodontal cells			
Stem cells from exfoliated deciduous teeth	Upregulation of angiogenic associated genes (KDR, SDF-1 and FGF2), osteogenesis-related genes (COL1, Runx2 and OPN), and phosphorylated AMP-activated protein kinase (p-AMPK) was observed	Enhanced osteogenesis and angiogenesis via AMPK signaling pathway	New blood vessels are formed and new bone is formed	Wu et al. (2019)
Human adipose-derived mesenchymal stem cells	Decreased levels of inflammation-related factors TNF-α, IL-1, IL-6	1. Inhibits inflammation-induced bone loss by reducing proinflammatory cytokine production	Inhibits inflammatory bone loss	Li et al. (2018a)
		2. Regulate the function of macrophages to regulate the inflammatory process		
Periodontal ligament stem cells (PDLSC)	Downregulation of TNF α and upregulation of IL-10, Arg-1 and CD163	The promotion of polarization toward an anti-inflammatory phenotype in non-activated and anti-inflammatory macrophages is facilitated	Enhanced periodontal regeneration	Liu et al. (2019)

Currently, implants represent a promising therapeutic modality for the treatment of periodontitis, with the potential to ameliorate alveolar bone resorption and enhance periodontal health. Research conducted by TAKAMASA Kawai et al. (2015) and others have revealed that the conditioned medium of bone marrow-derived mesenchymal stem cells (MSC-CM) contains various cytokines and chemokines, which can promote periodontal tissue regeneration through the mobilization of endogenous mesenchymal stem cells, angiogenesis, and differentiation processes. In their seminal study, Nagata et al. (2017) reported that transplantation of conditioned medium of human-derived periodontal ligament stem cells (PDLSC-CM) into rat mandibular defects enhances *in vivo* periodontal regeneration in a concentration-dependent manner. Moreover, they observed that PDLSC-derived conditioned media suppresses the mRNA expression of the inflammatory cytokine TNF-α in IFN-γ-stimulated RAW cells of the monocyte/macrophage lineage. Ma et al. (2022) demonstrated that extracellular vesicles derived from dental follicle stem cells (DSCs) promote periodontal tissue repair in experimental periodontitis by stimulating the proliferation, migration, and osteogenic differentiation of DSCs and promoting the formation of new periodontal ligament-like structures and bone. Their findings further revealed that these regenerative effects were partially mediated by the activation of the P38 MAPK signaling pathway. Nakao et al. (2021) utilized tumor necrosis factor-alpha (TNF-α)-pretreated gingival mesenchymal stem cells (GMSCs) to obtain pretreated exosomes and investigated their therapeutic effect on periodontal disease. The study found that exosomes derived from TNF-α-pretreated GMSCs can reduce periodontal bone resorption by upregulating CD73 and miR-1260b to regulate inflammatory responses, increasing CD73 expression, and inducing M2 macrophage polarization. Moreover, exosomal miR-1260b was identified as a key regulatory factor in preventing inflammatory bone loss by inhibiting the process of osteoclastogenesis through the Wnt5a-mediated RANKL pathway. In a murine model of periodontitis, dental pulp stem cells were found to induce a phenotypic shift in macrophages from a pro-inflammatory state to an anti-inflammatory state. This shift led to the inhibition of periodontal inflammation, the modulation of the immune response, and the promotion of healing of the alveolar bone and periodontal epithelium. The observed therapeutic effects of dental pulp stem cells may be attributed to the presence of miR-1246 within DPSC-derived exosomes (Shen et al., 2020). Activation of the IL-10/IL-10R pathway by M2-like macrophage-derived exosomes (M2-Exos) was found to stimulate osteogenic differentiation in bone marrow stromal cells (BMSCs), suppress the formation of osteoclasts in bone marrow-derived macrophages (BMDMs), and mitigate alveolar bone resorption in mice with periodontitis. These findings suggest that M2-Exos have therapeutic potential for the treatment of periodontal diseases (Chen et al., 2022). Exosomes from human gingival mesenchymal stem cells (GMSC-Exo) have been shown by Sun et al. (2022) to decrease the inflammatory response of periodontal ligament stem cells (PDLSCs) induced by lipopolysaccharide (LPS) by lowering NF-B signaling and Wnt5a expression. According to their research, GMSC-Exo may be used as a possible therapeutic agent to treat periodontitis by reducing inflammation by controlling the Wnt5a and NF-κB signaling pathways. By encouraging the growth of new periodontal tissue through immunomodulation and anti-inflammatory effects, exosomes derived from adipose-derived stem cells (adipose-derived stem cells-Exos) can be used as an adjunctive therapy for scaling and root planning (SRP), increasing the efficacy of non-surgical periodontal treatment (Mohammed et al., 2018). Exosomes derived from human exfoliated deciduous teeth (human exfoliated deciduous teeth-Exos) were found to ameliorate bone loss and promote osteogenic differentiation of bone marrow stromal cells (BMSCs) in a murine model of experimental periodontitis. These results suggest the potential therapeutic utility of human exfoliated deciduous teeth-Exos for the treatment of periodontal diseases (Wei et al., 2020). Moreover, exosomes isolated from human exfoliated deciduous teeth were shown to stimulate osteogenesis and angiogenesis via the activation of the AMPK signaling pathway, thus promoting periodontal bone regeneration. These findings suggest that exosomes derived from human exfoliated deciduous teeth may hold promise as a potential therapeutic strategy for the treatment of periodontal diseases (Wu et al., 2019). There are also reports on the use of exosomes derived from human exfoliated deciduous teeth with osteoinductive properties (SHED-Exos) to promote osteogenic differentiation of periodontal ligament stem cells (PDLSCs) through the Wnt/β-catenin and BMP/Smad signaling pathways (Wang et al., 2020). Studies have found that appropriate mechanical stimulation can help stem cells maintain their characteristics in an inflammatory microenvironment. Mechanical stress can induce exosomes from periodontal ligament stem cells to promote cell proliferation through the miR-181b-5p/PTEN/AKT signaling pathway and enhance osteogenic differentiation through BMP2/Runx2, thus maintaining the balance of the periodontal environment. The present study provides evidence that the newly identified physiological mechanical force can modulate the periodontal microenvironment by facilitating intercellular communication via exosomes in periodontal tissues. Furthermore, our findings suggest that exosomes induced by mechanical force may have therapeutic potential for the treatment of periodontitis and promotion of periodontal regeneration (Lv et al., 2020). Recent research has elucidated the mechanism by which exosomes derived from periodontal ligament stem cells (PDLSC-Exos) exposed to tensile stress regulate osteogenesis. Specifically, biophysical stimulation has been shown to enhance the osteogenic potential of PDLSC-Exos, thereby facilitating alveolar bone regeneration in periodontitis and offering a novel therapeutic avenue for the management of periodontal bone loss. Furthermore, it has been demonstrated that the miR200 family of bone marrow mesenchymal stem cell miRNAs serves as the primary mechano-responsive miRNA that initiates osteogenic differentiation (Wang et al., 2023). Based on these studies, we speculate that the osteogenic ability of exosomes can be enhanced through appropriate mechanical stimulation and other pretreatment methods, providing new directions for future research.

## 5 The possible mechanism of exosome-mediated periodontal tissue regeneration

### 5.1 Immunoregulation and inflammatory regulation of exosomes

Periodontitis is a chronic inflammatory illness defined primarily by overactivation of the host immunological response, which results in the stimulation of osteoclasts and subsequent alveolar bone loss. Periodontitis pathophysiology involves a variety of immune cells, including mononuclear phagocytes, antigen-presenting cells, particular T-cell subpopulations, B cells, and neutrophils. Exosomes possess immunomodulatory properties that facilitate periodontal tissue regeneration. It has been shown that Mesenchymal stem cell extracellular vesicles can promote bone regeneration through immunomodulatory effects (Kang M. et al., 2022). Mesenchymal stem cell exosomes stabilize the bone graft environment, ensure blood supply, promote osteogenic differentiation, and accelerate bone remodeling (Kang et al., 2022). The inflammatory bone loss caused by periodontitis may also promote periodontal tissue repair through the immunomodulatory effect of exosomes. Research indicates that the balance between helper T cells 17 (Th17) and regulatory T cells (Treg) is perturbed in the peripheral blood of patients with periodontitis. Notably, exosomes derived from periodontal ligament stem cells (PDLSC-exos) have been demonstrated to transport miR-155-5p to CD4^+^ T cells, resulting in altered SIRT1 protein expression and modulation of the Th17/Treg balance in periodontitis. These findings suggest that PDLSC-exos may have therapeutic potential for the management of periodontal diseases via their immunomodulatory effects (Zheng et al., 2019). The scientific literature suggests that extracellular vesicles originating from bone marrow mesenchymal stem cells may participate in the OPG-RANKL-RANK signaling pathway, thereby modulating osteoclast activity and impeding the progression of periodontitis and immune-mediated damage by modulating macrophage polarization and regulating inflammatory immune responses. These findings support the notion that extracellular vesicles from bone marrow mesenchymal stem cells may serve as a promising therapeutic modality for the treatment of periodontal diseases (Liu et al., 2021). TNF-α treatment of exosomes derived from gingival mesenchymal stem cells upregulates CD73 and miR-1260b, inducing macrophage polarization toward the M2 type, promoting inflammation resolution in periodontal tissues, and preventing further loss of alveolar bone (Nakao et al., 2021). Exosomes derived from pulp stem cells also induce macrophages in periodontitis mice to shift from pro-inflammatory phenotypes to anti-inflammatory phenotypes (Shen et al., 2020).

Furthermore, the modulation of key inflammatory cytokine expression in periodontal tissue is closely linked to the regeneration of periodontal tissues. Notably, conditioned media derived from human adipose-derived mesenchymal stem cells markedly decreases the mRNA expression levels of TNF-α, IL-1, and IL-6 in lipopolysaccharide-activated macrophages, indicating that AMSC-CM exerts a bone-protective effect by suppressing the production of pro-inflammatory cytokines. These results suggest that AMSC-CM may represent a promising therapeutic approach for the management of periodontal disorders (Li et al., 2018). The conditioned medium from periodontal ligament stem cells (PDLSC-CM) can also promote the differentiation of macrophages toward an anti-inflammatory phenotype by reducing the expression of tumor necrosis factor-alpha (TNF-α) and increasing the expression of interleukin-10 (IL-10), arginase-1 (Arg-1), and CD163 (Liu et al., 2019). SHED-derived exosomes were found to inhibit the expression of pro-inflammatory cytokines IL-6 and TNF-α (Wei et al., 2020). Following the administration of conditioned medium derived from periodontal ligament stem cells (PDLSC-CM), the mRNA expression level of TNF-α in periodontal tissues decreases, concomitant with a tendency toward reduced levels of COX-2, a molecule that is also implicated in inflammatory processes. These observations suggest that PDLSC-CM may exert anti-inflammatory effects on periodontal tissues, highlighting its potential as a therapeutic agent for the management of periodontal diseases (Nagata et al., 2017).

### 5.2 Promotion of endogenous stem cell regeneration and differentiation by exosome

Exosomes act as vectors for growth factors and cytokines that promote periodontal regeneration, trigger the proliferation of mesenchymal stem cells with the periodontal defects, and guide their differentiation into osteoblastic or odontoblastic lineages. The conditioned medium of bone marrow mesenchymal stem cells (MSC-CM) exhibits potent osteogenic capacity, which is mediated by the synergistic action of several growth factors, including IGF-1, VEGF, TGF-β1, and HGF (Inukai et al., 2013; Kawai et al., 2015). These cytokines regulate various osteogenic processes, including angiogenesis, cell migration, proliferation, and osteoblastic differentiation. IGF-1 plays an important role in matrix synthesis and mineralization during bone formation and promotes bone proliferation (Cornish et al., 2004) and migration (Li et al., 2007) of osteoblast-like cells. In addition, IGF-1 can stimulate PDL cell activity via the PI3K pathway, thereby enhancing periodontal regeneration (Han and Amar, 2003). VEGF (vascular endothelial growth factor) promotes endothelial cell survival and differentiation and is believed to have the capacity to directly orchestrate angiogenesis *in vivo*, thereby contributing to osteogenesis (Darnell et al., 2003). In skeletal tissues, TGF-β1 (transforming growth factor-β1) plays a crucial role in development and maintenance, simultaneously affecting both the osteoblastic and osteoclastic lineages and contributing to the dynamic equilibrium between bone resorption and formation (Janssens et al., 2005). Studies have demonstrated that transforming growth factor-β (TGF-β1) can regulate the proliferation, differentiation, and matrix formation of periodontal ligament (PDL) cells, thereby promoting regeneration and repair of the PDL (Gao et al., 1998; Tsuyoshi et al., 2004). HGF is also a potent angiogenic factor that primarily mediates its effects through direct action on endothelial cells. Therefore, exosomes can promote osteogenesis by carrying these growth factors and cytokines.

### 5.3 The role of exosomes in promoting angiogenesis in periodontal tissue regeneration

Angiogenesis plays an indispensable role in tissue repair and regeneration, and previous investigations have established a correlation between periodontal bone regeneration and angiogenesis. Notably, the conditioned media of bone marrow mesenchymal stem cells (MSC-CM) contain growth factors such as IGF-1, VEGF, and TGF-β1, which promote endogenous stem cell migration, proliferation, and tissue vascularization, thereby facilitating early bone formation and maturation in rabbits undergoing maxillary sinus lift surgery. These results suggest that MSC-CM may represent a promising therapeutic approach to enhance bone regeneration and tissue repair in the context of periodontal disease (Katagiri et al., 2015). The osteogenic differentiation of human gingival mesenchymal stem cells can be induced by conditioned media derived from human umbilical vein endothelial cells and bone marrow-derived mesenchymal stem cells, thereby promoting osteogenesis.

## 6 The advantages and limitations of exosomes in periodontal tissue regeneration

Exosomes present a promising alternative to traditional stem cell therapy in tissue regeneration and repair. First, exosomes possess reparative and regenerative properties similar to those of as their parental cells, including tissue repair, anti-inflammatory and immunomodulatory functions. Second, exosomes can be stably stored for extended periods due to their unique membrane structure, which protects them from enzymatic degradation, and they contain a rich array of bioactive molecules, including various growth factors, miRNAs, and protein molecules, which can enhance their therapeutic efficacy. However, the current route of administration for EVs is injection, which can result in rapid clearance by host cells and a short half-life *in vivo*. Studies have found that exosomes

Obtained from B16-BL6 cells are rapidly cleared from the circulation by the liver and subsequently delivered to the lungs (Takahashi et al., 2013). In periodontal tissue regeneration therapy, controlling the drug loading and release of exosomes is challenging, the overall regeneration performance is not satisfactory, and it is uncertain which proteins in exosomes have an effect on periodontal regeneration. Therefore, a key issue in utilizing exosomes as a cell-free therapy is to improve exosome retention at the site of injury. Furthermore, the inflammation-mediated bone resorption involved in periodontitis cannot be fully reflected in current experimental animal models.

## 7 Periodontal tissue engineering

Periodontal tissue engineering mainly includes three aspects: cell therapy, biomaterial scaffold fabrication technology, and bioactive factors (Poongodi et al., 2021). Biomaterial scaffolds have emerged as a promising strategy for tissue engineering and regenerative medicine applications. These scaffolds, designed to mimic the structures and functions of natural extracellular matrices, offer a novel approach to repair and regenerate damaged tissue. Their widespread use in these fields underscores the potential benefits of biomaterial scaffolds for promoting tissue regeneration and healing (Cheng et al., 2019). Ideal tissue engineering scaffolds should possess the following characteristics: good biocompatibility and biodegradation; controllable degradation and absorption rates; three-dimensional porous structures that support cell growth and transport of nutrients and metabolic waste; good plasticity and strength; mechanical properties that match the tissue at the implantation site (Padial-Molina and Rios, 2014) and minimal inflammation or toxicity *in vivo*. Existing scaffold materials include natural polymers, synthetic polymers, and bioceramics. The main types, advantages and functions of biomaterials used in periodontal tissue engineering are listed in Table 2. Natural polymer coatings encompass a variety of polysaccharides, including alginate, hyaluronic acid, chitosan, and chitin, as well as polymeric proteins, such as gelatin, collagen, silk fibroin, fibronectin, and keratin (Poongodi et al., 2021). The natural polymers mentioned above are characterized by their exceptional biocompatibility and bioactivity, which facilitate improved interactions between scaffold and tissue, as well as enhanced cell adhesion and proliferation. These properties ultimately support tissue repair and regeneration (de Queiroz Antonino et al., 2017). However, limitations such as rapid *in vivo* degradation and non-adjustable physicochemical properties have restricted their applications in the field of tissue regeneration (Sheikh et al., 2017). Artificial synthetic materials mainly include composite polymers such as polyglycolic acid (PGA), polylactic acid (PLA), and copolymer poly (lactic-co-glycolic acid) (PLGA) (Maeda et al., 2013). These materials are less costly to produce than natural polymers and can be structurally modified to meet treatment requirements such as the *in vivo* degradation rate, mechanical strength, and simple and straightforward manufacturing processes that allow mass production (d’Avanzo et al., 2021). Synthetic polymers are less biocompatible than natural polymers, which mainly limits their ability to support cell adhesion or migration, resulting in less efficient regeneration of damaged periodontal tissues. To overcome these limitations synthetic polymers are often utilized in combination with natural polymers (Kim et al., 2011). Bioactive ceramic materials, such as hydroxyapatite (HA), β-tricalcium phosphate (β-TCP), and bioactive glasses, have found extensive applications in promoting alveolar bone healing in periodontal tissues (Wang et al., 2022). Electrospun nanofibers with good biocompatibility possess inherent advantages, such as the ability to mimic the natural extracellular matrix (ECM), controllable degradation rates, and the ability to obtain excellent mechanical properties by adjusting relevant parameters. To address the diverse needs of periodontal regeneration, electrospun nanofibers can be modified with a variety of polymers and additives, including active bioceramics, growth factors, proteins, and drugs, to attain the desired properties. This adaptable approach enables the development of tailored scaffolds to address specific challenges in periodontal tissue engineering (Zhuang et al., 2019). The main advantage of 3D printing is that it sets parameters precisely at the stage of production, allowing the physical support of a 3D structure very similar to the extracellular matrix to be created under physiological conditions (Patel et al., 2017).

**TABLE 2:** The main types, advantages and effects of biomaterial scaffolds in periodontal tissue engineering.

Types of biomaterial scaffolds for periodontal tissue engineering	Included type	Advantages	Effects in the periodontal tissues
Natural polymers	Collagen, chitosan, alginate, fibrin, etc.	1. Good biocompatibility and low immunogenicity	1. 3D scaffold made of collagen hydrogel produced alveolar bone, dental bone and periodontal membrane reconstruction in Beagle dogs with class II bifurcation defects Kosen et al. (2012)
		2. Achieve better scaffold-tissue interaction, cell adhesion, proliferation and eventual tissue repair	2. Anti-inflammatory and antibacterial drugs encapsulated in chitosan hydrogel formed a dual-action system to treat periodontitis through anti-inflammatory and antibacterial effects, which effectively alleviated alveolar bone loss and suppressed local inflammatory response in rats with periodontitis Aminu et al. (2019); Ma et al. (2022b)
			3. The application of platelet-rich fibrin is shown to facilitate tissue-specific enhancement of alveolar bone and regeneration of periodontal soft tissues in the surrounding area Li et al. (2013)
Synthetic polymers	Polylactic acid, polyglycolic acid, poly (lactic-co-glycolic acid) (PLGA) and polycaprolactone (PCL)	1. Highly adjustable physicochemical properties	1. Novel hydroxyapatite (HAp) nanowire modified poly (lactic acid) (PLA) membranes with dual barrier/osteogenic induction function to promote bone repair and regeneration Han et al. (2018)
		2. Highly controllable biodegradation rate	2. Three-dimensional scaffolds prepared by electrospun polycaprolactone/gelatin (PCL/Gel) fiber membranes promote periodontal tissue regeneration in a rat model of acute periodontal defects Xu et al. (2022)
		3. A facile and streamlined manufacturing process suitable for large-scale production	
Bioceramics	Hydroxyapatite (HA), β-tricalcium phosphate (β-TCP), bioactive glass, etc.	1. High mechanical stability	1. Chitosan gel and hydroxyapatite powder inhibited the production of pro-inflammatory cytokines and increase BMP-2 production in periodontal tissue regeneration Gani et al. (2022)
		2. Excellent osteoconductivity and osteoinductivity suitable for alveolar bone regeneration	2. Dental grafting with β-tricalcium phosphate scaffolds accelerated bone formation and periodontal tissue regeneration Uchikawa et al. (2021)
			3. Bioactive glass/nano-hydroxyapatite is a good performance material in guided tissue regeneration Sunandhakumari et al. (2018)

In the realm of periodontal tissue engineering, bioactive factors have been extensively investigated, wherein polypeptide proteins such as platelet-derived growth factor (PDGF), bone morphogenetic proteins (BMPs), basic fibroblast growth factor (bFGF), and concentrated growth factor (CGF) have emerged as prominent examples. These factors exhibit a broad range of functions, including the stimulation of cell growth, proliferation, differentiation, and gene expression, thereby facilitating the functional restoration of periodontal tissue (Thoma et al., 2017; Kavyamala et al., 2019). Typically, cell therapy involves transferring new cells into tissues to treat disease. In regenerative medicine, cells are often used to improve the regeneration process by promoting the growth and differentiation of new tissue (Mao et al., 2006). To achieve optimal periodontal regeneration, transplanted cells into the periodontal defect should possess certain characteristics, including ease of acquisition, non-immunogenicity, high proliferative capacity, and the ability to differentiate into various cell types that comprise periodontal tissue (Fang et al., 2015). Recent advancements in tissue engineering utilizing mesenchymal stem cells (MSCs) have opened up new therapeutic avenues for the repair and regeneration of tooth and periodontal tissues. Stem cells used for periodontal regeneration can be classified into dental and non-dental stem cells. A variety of stem cell types, such as dental pulp stem cells (DPSCs), stem cells derived from human exfoliated deciduous teeth (SHED), periodontal ligament stem cells (PDLSCs), stem cells from the apical papilla (SCAPs), and dental follicle cells (DFCs), have demonstrated significant potential in the regeneration of dental tissue (Zhai et al., 2019). Furthermore, several other stem cell types, including embryonic stem cells, induced pluripotent stem cells, bone marrow-derived mesenchymal stem cells, and adipose-derived stem cells, have also exhibited the ability to enhance periodontal tissue regeneration.

In this section, we summarize the main biomaterials used in periodontal tissue engineering, including natural polymers, synthetic polymers and bioceramics, and discuss their advantages and disadvantages. In general, these materials can promote the reconstruction and regeneration of alveolar bone, cementum and periodontal ligament to some extent. We hope that the present problems and challenges can be overcome through the improvement of synthetic technology in the future, in order to realize the wide application of biomaterials in periodontal tissue engineering.

## 8 Combination of exosomes and scaffold

For a biological scaffold loaded with exosomes, it is necessary to effectively retain the exosomes at the implantation site while preserving their activity and ensuring sustained release. Extensive research has been conducted on the characteristics of scaffolds with different materials and structures that can provide safe and stable carriers for exosome delivery *in vivo*. Currently, composite techniques mainly include chemical crosslinking, physical adsorption, freeze-drying, 3D printing, and specific binding. Shafei et al. (2019) prepared alginate (Alg) hydrogels through ion crosslinking reactions, and added exosomes to the Alg solution, which was then mixed with calcium chloride (CaCl_2_) solution to form Alg hydrogels encapsulating exosomes. The degradation and cell viability of the exosome-containing hydrogels on the Alg hydrogel confirmed that the composite material used has suitable *in vivo* and *in vitro* application characteristics. Guan et al. (2022) introduced aldehyde-functionalized chondroitin sulfate (OCS) into gelatin methacryloyl (GM) to form a GMOCS hydrogel. The GMOCS mixtured solution, exosomes, and photoinitiator 2-hydroxy-1-[4-(hydroxyethoxy) phenyl]-2-methyl-1-propanone were mixed together, ensuring that the exosomes were uniformly distributed in the solution. Finally, the GMOCS-Exos gel was obtained by photocrosslinking under ultraviolet light. Zhu et al. (2022) produced Gel by dissolving gelatin in an LAP solution, soaking it in water bath at 55°C and adding exosomes to the mixture at room temperature. The mixture solution was then cross-linked under UV light. This hydrogel has been shown to significantly improve wound healing. Fan et al. (2022) developed a A double-mesh conductive hydrogel consisting of a photocrosslinkable gelatin methacrylate (GM) hydrogel and a polypyrrole (PPy) hydrogel. The GM hydrogel network was formed by photolinking GM under UV irradiation. The GM hydrogel was then sequentially immersed in a solution containing monomer Py and tannic acid (TA) and a solution containing ammonium persulfate (APS) for *in situ* polymerization and cross-linking of the conductive PPy chains. Finally, bone marrow mesenchymal stem cell-derived exosomes were immobilized onto the GM hydrogel network, forming a GMPE hydrogel through the reversible interaction between the abundant polyphenolic groups in TA and the phosphoric groups in exosomal phospholipids. This exosome-combined conductive hydrogel promoted neuron and axon regeneration in an SCI mouse model. Qi et al. (2016) utilized a highly porous, small-pore-sized, classical porous β-tricalcium phosphate (β-TCP) scaffold as an exosome carrier. Under sterile conditions, an equal amount of mesenchymal stem cell-derived exosomes from induced pluripotent stem cells (iPSC-MSC-Exos) was dripped onto each β-TCP scaffold, followed by freeze-drying. The resulting hiPSC-MSC-Exos+β-TCP scaffold was applied for bone regeneration in cranial defects. Liu et al. (2021) produced a hierarchically mesoporous bioactive glass (MBG) scaffold with macro/micro/meso-porous structures via freeze-drying. Mesenchymal stem cell osteogenic exosomes (BMSC-OI-exo) were then delivered to the hierarchically mesoporous bioactive glass (MBG) scaffold through lyophilization, achieving sustained and controlled release of the biological activity by embedding the exosomes in the micro-pores on the surface of the scaffold. Shi et al. (2017) dissolved chitosan in acetic acid solution, and then the resulting chitosan solution was mixed with silk fibroin solution. The mixture was freeze-dried in a vacuum freeze-dryer to produce a chitosan/silk hydrogel sponge. Finally, gingival mesenchymal stem cell-derived exosomes) were inserted into the chitosan/silk hydrogel sponge to observe their effects on skin defects in diabetic rats. Kyung Kim et al. (2021) also used the freeze-drying method to prepare silk fibroin scaffolds and coated them with isolated exosomes. The exosomes wrapped on the scaffold were not affected by any chemical treatment or synthetic program, which accelerated the osteogenic cell process regulated by exosomes in the rat cranial defect area. Chen et al. (2019b) designed a 3D biological scaffold for mesenchymal stem cell-derived exosome delivery. They used stereolithography to create 3D cartilaginous extracellular matrix (ECM)/methacrylic acid gel GelMA/*in vitro* scaffolds with radial guiding channels. This ECM/GelMA/Exosome scaffold can effectively repair chondrocyte mitochondrial dysfunction and promote chondrocyte migration. Hu et al. (2021) utilized cryogenic 3D printing to fabricate an acellular small intestinal submucosa (SIS) scaffold, which was combined with mesoporous bioactive glass (MBG) and exosomes to create a 3D scaffold dressing (SIS/MBG@Exos) capable of sustained release of bioactive exosomes. The resulting scaffold possessed a favorable 3D structure, appropriate porosity, biocompatibility and hemostatic ability, and was found to promote the proliferation, migration, and angiogenesis of human umbilical vein endothelial cells (HUVECs). Specific binding, which is typically regarded as a more convenient and feasible method for biomolecule attachment compared to traditional physical adsorption or chemical modification, was employed for biomolecule connection. A novel bio-specificity peptide (BSP) was designed by Zhang et al. (2021) to specifically bind to collagen scaffolds and human umbilical cord mesenchymal stem cell exosomes, respectively, finally, the linear and orderly collagen scaffold and paclitaxel (PTX) were constructed into a multifunctional collagen scaffold to repair spinal cord injury in rats. Zha et al. (2021) combined engineered exosomes with a 3D-printed porous bone scaffold made of polycaprolactone. The principle was to specifically bind the exosomes to the tetraspanin antigen CD63 enriched on the exosome surface using the exosome-anchoring peptide CP05. Therefore, modifying the 3D-printed scaffold with CP05 as an elastic linker could promote the transplantation efficiency. Zhang et al. (2016) used a physisorption technique to load human induced pluripotent stem cell-derived mesenchymal stem cell exosomes (HIPS-MSC-Exos) onto classical porous tricalcium phosphate scaffold material (β-TCP) for the treatment of bone defects. Similarly, Li et al. (2018) modified poly (lactic acid-glycolic acid) (PLGA) scaffolds by poly (dopamine) (PDA)-mediated immobilization of bone-forming peptide-1 (BFP-1) by infiltrating PLGA/PDA with exosome solution at low temperature to form a composite scaffold with PLGA/PDA physisorbed to Exosome.

In general, exosome composite scaffolds can be fabricated by different composite technologies, and the technology is mature. Different composite scaffolds have their own advantages, but all of them have appropriate porosity, good biocompatibility, and the ability to release exosomes continuously *in vivo* and maximize the retention of their activity. Therefore, the selection of a composite technology that is more suitable for periodontal application and has more bone repair advantages may be the key to translational application.

## 9 Application of exosome composite scaffold in periodontal regeneration

**FIGURE 2:**
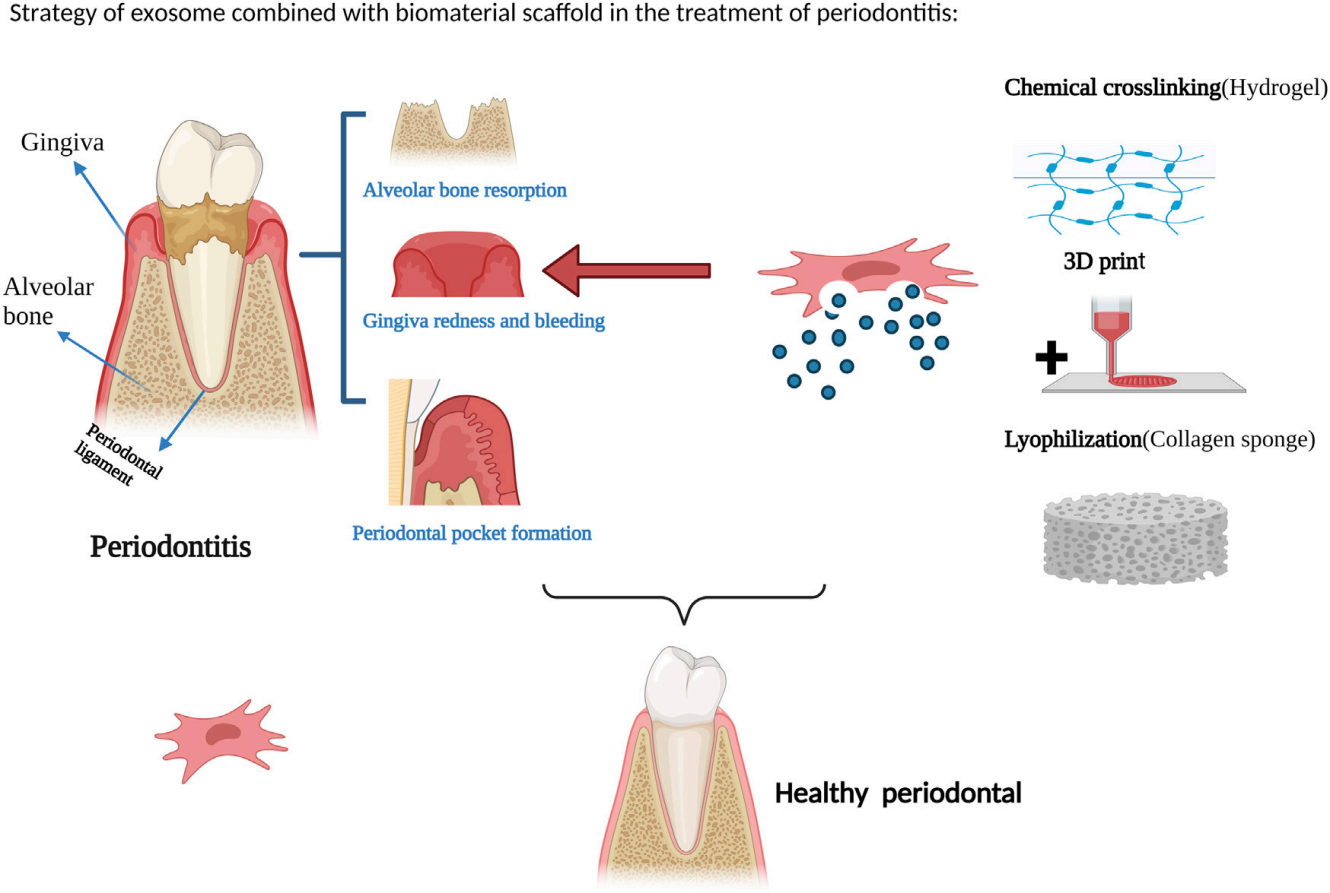
Strategy of exosome combined with biomaterial scaffold in the treatment of periodontitis. Figures created with BioRender.com with an academic license.

At present, research on the combination of exosomes and biomaterial scaffolds for tissue repair and regeneration has made significant progress and achieved certain therapeutic effects. Figure 2 illustrates a strategy for the treatment of periodontitis with exosomes in combination with biomaterial scaffolds. However, due to the limitations of the short half-life of exosomes *in vivo* and the inability to achieve local sustained release, exosome-combined scaffolds are gradually being introduced into the field of periodontal tissue engineering, with the aim of achieving periodontal tissue regeneration. Chew et al. (2019) evaluated the efficacy of a collagen sponge composite scaffold incorporating human bone marrow mesenchymal stem cell-derived exosomes in a rat model of periodontal defects. The authors observed that this scaffold facilitated the regeneration of periodontal tissue and alveolar bone without any adverse effects. The promotion of periodontal regeneration via exosome-mediated mechanisms may be attributed to the activation of adenosine receptors along the AKT and ERK signaling pathways, which in turn stimulate the migration and proliferation of periodontal ligament cells. Lei et al. (2022) used a composite scaffold of exosomes from of healthy periodontal ligament-derived periodontal ligament stem cells (h-PDLSCs) combined with Matrigel or β-TCP to repair bone defects in a rat model of periodontitis, and the results promoted regeneration of alveolar bone. Wu et al. (2019) used a β-tricalcium phosphate (β-TCP) scaffold loaded with exosomes derived from human deciduous teeth to treat periodontal defects in rats, resulting in the promotion of periodontal bone regeneration. Liu et al. (2021) injected hydrogels encapsulated with bone marrow mesenchymal stem cell extracellular vesicles (BMSC-EVs) into periodontal tissues of rats with experimental periodontitis, and the exosomal hydrogels were able to inhibit the development of periodontitis and immune damage by modulating the inflammatory immune response. The osteogenic potential of exosomes derived from periodontal ligament stem cells (PDLSC-Exos) and the efficacy of composite hydrogels loaded with PDLSC-Exos for the repair of alveolar bone defects in rats were investigated by (Wang R. et al., 2020). The composite hydrogel was prepared by mixing gelatin and sodium alginate in equal proportions and dissolving them in sterile calcium phosphate, cross-linking them with sterilized CaCl2 solution and removing CaCl2, and then mixing the exosomes into the Gel-Alg hydrogel. The results showed that the hydrogel successfully applied the exosomes from periodontal ligament stem cells for the early repair of alveolar bone defects in rats. Also demonstrated that chitosan hydrogels loaded with dental pulp stem cell exosomes promoted healing of alveolar bone and periodontal epithelium in mice with periodontitis. Inukai et al. (2013) created a critical-sized intra-bony periodontal defect in the mandible of dogs and used an absorbable collagen sponge as a scaffold material loaded with mesenchymal stem cell-conditioned medium (MSC-CM), and found that MSC-CM enhanced periodontal regeneration. Kawai et al. (2015) also combined MSC-CM with a collagen sponge for rat periodontal tissue defects, and found that the MSC-CM group showed periodontal tissue regeneration after 4 weeks of implantation.

All of the above studies have demonstrated that exosome composite scaffolds have shown good results in promoting periodontal tissue regeneration, which to a certain extent compensates for the practical application of exosomes such as the difficulty of controlling exosome loading and release, etc. There is still a need for further clinical studies to ensure that these materials can be practically used in patients.

## 10 Future directions and challenges

Exosomes represent a promising novel therapeutic tool for periodontal regeneration. The utilization of stem cell-derived exosomes for the repair of periodontal tissue defects has yielded preliminary positive outcomes, and exhibits certain advantages. This review provides an overview of the latest research developments in this field. First, in comparison to seed cells, exosomes exhibit superior biocompatibility, biodegradability, low immunogenicity, and an absence of ethical issues, rendering them safer and more practical. Second, a plethora of *in vitro* studies have demonstrated that exosomes participate in the transmission of inflammatory signals and the progression of periodontitis, thus furnishing potential therapeutic targets for periodontal regeneration (Zhao et al., 2019; Zheng et al., 2019; Wang et al., 2020). However, numerous issues still require immediate resolution. First, the extraction and purification techniques for exosomes are limited, with ultracentrifugation remaining the preferred method for isolation, despite its drawbacks of being time-consuming, labor-intensive, low in purity, and susceptible to degradation. Although new exosome extraction kits have been developed to increase yield, purity reductions have been observed. The preparation of high-purity, high-yield exosomes is a fundamental bottleneck for their application in tissue repair and necessitates the development of new separation techniques to meet clinical needs. Second, the absence of a global consensus agreement on exosome collection for tissue regeneration remains a challenge. Different cell types, culture conditions, and exosome batches can all have varying exosome contents, making it difficult to standardize exosome applications. Third, exosomes themselves exhibit low retention rates and short half-lives, which can lead to rapid metabolism *in vivo* and impact their functional efficacy.

At present, the integration of tissue engineering techniques and exosomes represents a viable approach for achieving sustained exosome release in tissue repair. A multitude of exosome-loaded scaffolds have been employed in preclinical investigations of tissue repair and regeneration, spanning diverse tissue types such as bone, cartilage, and skin, among others, revealing substantial therapeutic potential. In the field of oral tissue engineering, exosome-loaded biomaterial scaffolds can synergistically contribute to periodontal tissue regeneration, exhibiting excellent biological and physicochemical properties. Currently, the scaffolds used for loading exosomes in periodontal regeneration mainly include hydrogels, collagen sponges, and β-tricalcium phosphate (β-TCP) scaffolds, which provide more effective protection and fixation for exosomes, enable sustained delivery of exosomes, prevent premature elimination of exosomes by tissues, and promote their concentration release at the injury site or nearby. These exosome composite scaffolds have opened up broad prospects for their application in periodontal regeneration. Although the application of exosomes in periodontal regeneration has been proven effective in preclinical animal models, much work still needs to be done to apply them to clinical practice. The limitations currently encountered are mainly twofold: First, the inflammation-mediated bone resorption that is involved in periodontal defects resulting from periodontitis is far more complex than that observed in animal models currently established, and these animal models cannot fully reflect the chronic inflammatory process of periodontitis in clinical patients. Second, although some degree of periodontal tissue regeneration was observed in the current research, the overall regeneration level was is not satisfactory. Additionally, controlling the drug loading and release of exosomes in current exosome-composite scaffolds is extremely challenging. Therefore, to better investigate the therapeutic effect of exosomes on alveolar bone defects caused by periodontitis, further exploration of the immunomodulatory role of exosomes is needed in large animal models of periodontitis; the mechanisms underlying the dose-dependent effect of exosomes need to be studied; and the optimal tissue regeneration scaffold material and further safety investigations need to be determined, with the impact of exosomes monitored over an extended follow-up period.

## 11 Conclusion

Studies have shown that stem cells influence the biological behavior of cells in the microenvironment by releasing exosomes and other vectors through paracrine mechanism to mediate cell-to-cell communication and activate the signaling pathways of target cells, induction of regeneration of defective tissue. In this review, we discuss the application of exosomes in the treatment of periodontitis, either alone or in combination with tissue engineering techniques, which has achieved preliminary results and some advantages in the repair of defective periodontal tissues. As exosomes are more stable, Operability and widely available than conventional therapies, they have a wide range of potential applications. However, most studies on exosomes have been limited to animal models and have not applied them clinically to repair and regeneration of human oral tissues, and data samples are insufficient; The systematic mechanism of diagnosis and treatment in chronic periodontitis has not been effectively explained, and different doses of exosomes have different effects on bone regeneration and repair. To compare the effects of exosomes derived from different kinds of cells in inducing cementum regeneration, it is important to obtain the characteristics of functional cementum, periodontal ligament and alveolar bone, and to clarify the mechanism of exosome-induced regeneration. It is believed that exosomes combined with tissue engineering can provide new ideas for periodontal tissue regeneration, and exosomes will have more satisfactory research results in the field of regenerative medicine.
